# Antioxidant, Antimicrobial Activity and Toxicity Test of *Pilea microphylla*

**DOI:** 10.1155/2010/826830

**Published:** 2010-06-20

**Authors:** Amir Modarresi Chahardehi, Darah Ibrahim, Shaida Fariza Sulaiman

**Affiliations:** ^1^Industrial Biotechnology Research Laboratory, School of Biological Sciences, Universiti Sains Malaysia, Penang, 11800 Minden, Malaysia; ^2^Phytochemistry laboratory, School of Biological Sciences, Universiti Sains Malaysia, Penang, 11800 Minden, Malaysia

## Abstract

A total of 9 plant extracts were tested, using two different kinds of extracting methods to evaluate the antioxidant and antimicrobial activities from *Pilea microphylla* (Urticaceae family) and including toxicity test. Antioxidant activity were tested by using DPPH free radical scavenging, also total phenolic contents and total flavonoid contents were determined. Toxicity assay carried out by using brine shrimps. Methanol extract of method I (ME I) showed the highest antioxidant activity at 69.51 ± 1.03. Chloroform extract of method I (CE I) showed the highest total phenolic contents at 72.10 ± 0.71 and chloroform extract of method II (CE II) showed the highest total flavonoid contents at 60.14 ± 0.33. The antimicrobial activity of *Pilea microphylla* extract was tested in vitro by using disc diffusion method and minimum inhibitory concentration (MIC). The *Pilea microphylla* extract showed antibacterial activity against some Gram negative and positive bacteria. The extracts did not exhibit antifungal and antiyeast activity. The hexane extract of method I (HE I) was not toxic against brine shrimp (LC50 value was 3880 *μ*g/ml). Therefore, the extracts could be suitable as antimicrobial and antioxidative agents in food industry.

## 1. Introduction

In recent decades, the essential oils and various extracts of plants have been of great interest as they have been the sources of natural products [1]. The most commonly used antioxidant at the present time are butylated hydroxyanisole (BHA), butylated hydroxytoluene (BHT), propylgallate (PG), and tert-butyl hydroxyl toluene (TBHQ) [2]. However, they are suspected of being responsible for liver damage and carcinogenesis in laboratory animals [3, 4]. Therefore, the development and utilization of more effective antioxidant of natural origin are desired [5].

The antioxidant activity of phenolic compounds is mainly due to their redox properties, which can play an important role in absorbing and neutralizing free radicals, quenching singlet and triplet oxygen, or decomposing peroxidase [6]. Therefore, an investigation of such antioxidative phenolic compounds in edible plants has been conducted to improve our understanding of their dietary value and potential benefits [7]. *Pilea microphylla* (PM) (family: Urticaceae) is being used as folk medicine to treat several allergies/wounds in and around Malaysia peninsular especially Penang island. It is reported to possess antibacterial activity [8], moderate antioxidant activity, and total phenolic content [9] and PM is also used for infertility, inflammations, and womb cleanser [10].

There is no enough report about its antioxidant and antimicrobial activities. The purposes of this study were to compare the extracting method together and to evaluate *Pilea microphylla* in Urticaceae family as new potential sources of natural antioxidant and antimicrobial activities.

## 2. Materials and Methods

### 2.1. Plant Material and Extraction

The whole part of* Pilea microphylla* were collected in Penang island from USM main campus (Universiti Sains Malaysia) in March 2008. Voucher specimens have been deposited at the Herbarium of the School of Biological Sciences, Universiti Sains Malaysia in April 2008. The plant materials were washed, dried, and grounded to small pieces. The first method (Method I) of extraction included the using of four solvents by following nonpolar to polar solvents (by using Soxhlet apparatus). In this method, dried powdered plant was extracted. The solvents used were hexane, chloroform, ethyl acetate, and methanol. The second method (Method II) included 5 solvents system (by using partition technique). For Method II, the dried materials were extracted by using soxhlet extractor with methanol as a solvent for 72 hours at room temperature (30°C). The methanolic extracts were further partitioned by adding distilled water in a separating funnel and then followed using chloroform, diethyl ether, ethyl acetate, and butanol as described by Mellidis et al. (1993), with a slight modification [11]. The dried extracts were then weighed using microbalance and were kept at 4°C. Abbreviations for crude extract used in this paper, namely, HE I (hexane extract of method I), CE I (chloroform extract of method I), EAE I (ethyl acetate extract of method I), ME I (methanol extract of method I), ME II (methanol extract of method II), CE II (chloroform extract of method II), DEE II (diethyl ether extract of method II), EAE II (ethyl acetate extract of method II) and BE II (butanol extract of method II).

### 2.2. Determination of Antioxidant Activity: DPPH Radical Scavenging Assay

Free radical scavenging activity of *Pilea microphylla* (MeOH, Chlorofom, Diethyl ether, Ethyl acetate, n-hexane, and Butanol extracts) were tested using DPPH method with final concentration 1000 *μ*g/mL (Etanolic DPPH) (Sigma Chemical Co., USA) (300 *μ*M) and was used in the reaction mixture. Freshly prepared test samples (50 *μ*L) were combined with DPPH solution (150 *μ*L) in a 96 well microtiter plate. DMSO was used as a negative control. The reaction mixture were incubated for 30 minutes at 37°C and the change in absorbance at 515 nm was measured using micro plate reader (Thermo Electron Corporation, Finland). All determinations were performed in triplicate. The obtained absorbance values were converted into the percentage of radical scavenging activity using the following equation:

(1) $$\text{Radical}\;\text{scavenging}\;\text{activity}\;\left(\text{\%}\right)=\;100-\left[\frac{\text{AS}}{\text{A}\;\text{C}}\times 100\right],$$

where AS: absorbance of the sample, AC: absorbance of the negative control.

Free radical scavenging potency as determined from EC_50_ value obtained. Lower EC_50_ value indicated strong free radical scavenging activity.

### 2.3. Determination of Total Phenolic Compounds

The amounts of phenolics in the selected medicinal plant extracts were determined with Folin-Ciocalteu reagent using the method of Salvi et al. a little bit modified [12].0.5 mL of each sample was dissolved in 1.0 mL DMSO (3 replicates), 1.0 mL of 10% dilution of Folin-Ciocalteu reagent and after 3 minutes, 3 mL of Na_2_CO_3_ (1%, w/v) were added and the resulting mixture was incubated at room temperature for 2 hours. The absorbance of all samples was measured at 760 nm using a spectrophotometer(Model U-1900 Spectrophotometer Hitachi High Technology Corporation 2006).The standard curve was prepared using 0, 50, 100, 150, 200, and 250 mg/l. Results were expressed as milligrams of gallic acid equivalent per gram of dry weight (mg GAE/g dry weight), which is a common reference compound.

### 2.4. Determination of Total Flavonoid Content

The total flavonoid content was determined using the Dowd method [13]; 5 mL of 2% aluminium trichloride (AlCl_3_) in methanol was mixed with the same volume of the extract solution (0.4 mg/mL). Absorption readings at 415 nm using PerkinElmer UV-VIS lambda 25 spectrophotometer were taken after 10 minutes against a blank sample consisting of a 5 mL extract solution with 5 mL methanol without AlCl_3_. The total flavonoid content was determined using a standard curve with quercetin (0–100 mg/l) as the standard. Total flavonoid content is expressed as mg of quercetin equivalents (QE)/g of extract.

### 2.5. Antimicrobial Activity Test

Antimicrobial activity was determined using Disc Diffusion following the method described by National Committee for Clinical Laboratory Standard (NCCLS) [14]. All bacterial strains including clinical and ATCC were used in the study. The test bacteria was removed aseptically with an inoculating loop and transferred to a test tube containing 5 mL of sterile distilled water. Sufficient inocula were added until the turbidity equaled 0.5 McFarland (10^8^ cfu/mL) standards (bioMerieux, Marcy d'Etoile, France). The test tube suspension (1 mL) was added to 15–20 mL of nutrient agar or Sabouraud dextrose agar before setting aside the seeded agar plate (9 cm in diameter) to solidify for 15 minutes. Three disks of Whatman's No. 1 filter paper, 6 mm in diameter, were used to screen the antimicrobial activity. Each sterile disk was impregnated with 20 *μ*L of extract (corresponding to 100 mg crude extract/mL), amoxicillin (and vancomycin for *Streptococcus sp*.), miconazole nitrate (30 *μ*g/mL, as positive control for bacteria and fungi, respectively), or 10% DMSO (v/v) (as negative control), before it was placed on the surface of the seeded plates. The plates were incubated at 37°C overnight and examined for zones of growth inhibition. Antibacterial activity was recorded if a zone of growth inhibition around the well of greater than 6 mm was measured. Any extracts showing inhibition was tested twice more.

### 2.6. Determination of the Minimum Inhibitory Concentration (MIC)

MIC was determined by the liquid dilution method. Dilution series were set up with 0.13, 0.26, 0.52, 1.04, 2.10, 4.17, 8.33, 16.66, 33.33, 66.66, and 133.33 mg/mL of nutrient broth medium. To each test tube, 0.5 mL of standardized suspension of bacteria were added and incubated at 37°C for 24 hours. The tests were continued out in triplicates. The lowest concentration which did not show any growth of the tested microorganism after macroscopic evaluation was determined as the MIC.

### 2.7. Determination of the Minimum Bactericidal Concentration (MBC)

The minimum bactericidal concentration of the plant extract on the clinical bacterial isolates was done according to the method highlighted in National Committee for Clinical Laboratory Standard (2002). Briefly, 1 mL that was pipetted from the mixture obtained in the determination of MIC stage was streaked out on the nutrient broth for 24 hours. The least concentration of the extract with no visible growth was taken as the minimum bactericidal concentration.

### 2.8. Toxicity Assay

The preliminary biological evaluation of the crude extract of *Pilea microphylla* was determined using brine shrimp lethality test according to Simionatto et al. with some modification [15]. The brine shrimp (*Artemia salina*) lethality assay is considered to be a useful tool for preliminary assessment of cytotoxicity [16]. Brine shrimp assays have also been used for the analysis of pesticides residues [17], to monitor the toxicity of organic waste to marine organisms [18] and active plant constituents [19]. The Brine shrimp eggs were provided by Muka Head Marine Research Station of the Universiti Sains Malaysia, and were hatched in artificial sea water (38 g salt per liter of water). After 24 hours, the hatched nauplii suspension was left to stand for 1 hour without aeration, and then the nauplii were collected by pipetting from middle layer of solution, in which most of nauplii were swimming. The crude extracts were dissolved in distilled water to various concentrations and the shrimp larvae were placed in to them (duplicate). Sea water without extract was used as a negative control, while potassium dichromate had a LC_50_ = 20 *μ*g/mL as a positive control. Fifteen nauplii were withdrawn through a glass capillary and placed in each vial containing 4.5 mL of brine solution. Final concentration of extracts in each experiment were used 5.000, 2.500, 1.250, 0.625, 0.312, 0.156, and 0.078 mg/mL. 0.5 mL of the plant extract was added to 4.5 mL of brine solution and maintained at room temperature for 24 hours under the light and surviving larvae were counted.

### 2.9. Statistical Analysis

Results were tested for statistical significance by GraphPad Prism. Differences were considered statistically significant at the *P* < .05 level and One Way ANOVA.

## 3. Results

### 3.1. Antioxidant Activity

The results are shown in Table 1. ME I showed the highest antioxidant activity of 69.51 ± 1.03% with the lowest EC_50_ of 81.32 *μ*g/mL, if compared to other extracts. Thus, the extracts were used to screen for the radical scavenging activity at different concentrations. A narrow range of total phenolics content was found in study. Their content ranged from 9.91 ± 0.33 to 72.10 ± 0.71 mg gallic acid equivalent (GAE)/g of extracts, with an average of 41.00 mg/g. As shown in the Table 1, CE I had the highest phenolic at 72.10 ± 0.71 mg GAE/g and CE II had the highest flavonoid contents at 60.14 ± 0.33 mg QE/g, unless CE I showed higher flavonoid contents after CE II at 53.44 ± 0.94 mg QE/g.

CE I and EAE I showed the weakest DPPH radical scavenging capability, at only 0.46 ± 0.98 and 4.10 ± 0.14, respectively. For further study, butanol extract of method II (BEPM II) was selected for get fractionations by paper chromatography (PC). Four bands were obtained. BEPMf_4_ displayed the highest antioxidant activity than other fractions (27.84 ± 1.27). 

Generally, if the ratio between total phenolic content and total flavonoid as shown would be more than 1, it showed an increase at in total phenolic content of the extract (as shown in last column). The relationship between total phenolic content and antioxidant activity of PM was calculated in both methods of extraction. The results indicated that when all extracts in method II were included in the statistical analysis, there was a negative relationship between them (*R* ^2^ = 0.051), unless method I showed a little bit correlation between the antioxidant activity and total phenolic contents (*R* ^2^ = 0.636).

### 3.2. Antimicrobial Activity

The antimicrobial effects of PM extracts on pathogenic bacteria, fungi and yeasts are presented in Table 2. In addition, pure methanol (control) had no inhibitory effects on pathogenic microbes tested. The PM extracts at 100 mg/mL concentration were effective on the tested bacteria. PM extracts gave large inhibition zone on *Bacillus spizizenii* ATCC 6633 and *Micrococcus sp.* by using CE I and CE II, respectively. According to Table 2, CE II was effective against on *Escherichia coli* and methicillin resistant *Staphylococcus aureus* (MRSA).

All tested extracts showed no antifungal activity except hexane extract against *Aspergillus niger USM AI1 *showed reduced of UFC, but nor hypha.

The MIC and MBC values that were tested on bacteria are listed in Table 3. The MBCs were defined as the lowest concentration that killed at least 99.9% of the cells in the initial inoculum. The MBCs were tested for which has shown high antimicrobial in MIC test.

MIC values range between 8.33–33.33 mg/mL. EAE I showed the lowest concentration of MIC and MBC values of 8.33 mg/mL against *Bacillus spizizenii *ATCC 6633. 

### 3.3. Toxicity Test

The best extracts from antimicrobial activity were selected for toxicity test. Brine shrimp results presented in (Table 4) show that HE I and BE II were virtually nontoxic on shrimps, and also they exhibited very low toxicity, giving LC_50_ values greater than 100 *μ*g/mL after 24 hours.

**Figure 1:**
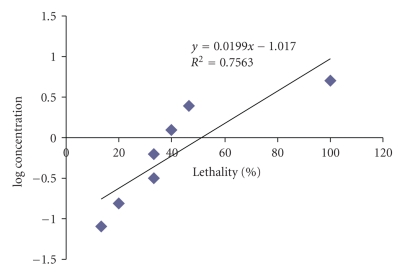
Toxic effects of the *P. microphylla *hexane extract of method I after 24 hours using brine shrimp lethality assay.

There is a wide range of LC_50_ values of crude extracts between 2–3880 *μ*g/mL. The hexane extract of method I (LC_50_ 3880 *μ*g/mL) exhibited very low toxicity, (Figure 1).

## 4. Discussion

*Pilea microphylla *is used in traditional medicine to treat bacterial infection [8].The claims for treatment infertility, inflammation and cleaning of reproductive sex organ of female human [10].

The results of this screening experiment demonstrated that *P. microphylla* contained different levels of total phenolic, total flavonoid and possessed diverse antioxidant properties. Methanol extract of method I showed the highest antioxidant activity with low EC_50_. Also, previous study showed an ethanolic extract of *Pilea microphylla* which was found to be most potent when subjected to detailed free radical scavenging at 23.15 *μ*g/mL [20]. Otherwise, methanol and ethanol are in same polarity level. Our results confirmed their results.

In this study, *Pilea microphylla* had a variety of antimicrobial activity against pathogenic microorganisms. The majority of these crude extracts which inhibited growth of bacteria were active against Gram-positive bacteria such as *B. cereus*, *B. subtilis,* and Methicillin resistant *Staphylococcus aureus, *unless both nonpolar and polar extracts in method II and butanol extract as a polar extract in method I, exhibited low activity against *Bacillus cereus *and *Bacillus cereus *ATCC 10876. The results seem to indicate that method II (sequential partitions) may be more effective than method I (by using soxhelt extractor). *E. coli*, *B. subtilis*, Methicillin resistant *Staphylococcus aureus* (MRSA) inhibited by extracts from method II. This reason may be related to the extracting method from polar to nonpolar components. This study has shown that 63.6% of extracts inhibited Gram-positive bacteria (7 out of 11) and 60% of extracts also inhibited Gram-negative bacteria (9 out of 15). However, the frequency of antimicrobial activity statistically was 13.13% (39 out of 297). Facey et al. found low antibacterial activity against *Staphylococcus aureus *by using mixture of acetone and ethyl acetate of *P. microphylla* [8].

The greater resistance of Gram-negative bacteria to plant extracts has been documented previously [21, 22] and is supported by our results. These observations are likely to be the result of the differences in cell wall structure between Gram-positive and Gram-negative bacteria, with the Gram-negative outer membrane acting as a barrier to many environmental substances, including antibiotics [23]. The disc diffusion method showed MIC values of 8.33 to 33.33 mg/mL. The PM extracts also could be used to treat most of *Bacillus sp., Escherichia coli, *and methicillin resistant *Staphylococcus aureus* (MRSA). 

Hexane extract was not toxic against brine shrimp (LC_50_ value more than 1 mg/mL). This is impressive and lends support to the suggestion that hexane extract of PM can be used to treat pathogenic microbial infections such as *B. cereus* that can cause food poisoning symptoms.

In conclusion, *Pilea microphylla* could be suggested as a natural source of antioxidant and antiaging factor. Further evaluation of the antibacterial properties of its extracts and elucidation of the components responsible for the activities is warranted. Two of the organisms inhibited by some of the extracts, *B. cereus* and MRSA, are important food-borne pathogens, raising the possibility of using the active compounds of this plant to prevent food-borne diseases. However, the application of any compounds to medicine will require safety and toxicity issues to be addressed and further study for purification of the active compound needs to be done for *Pilea microphylla*.

## Figures and Tables

**Table 1:** List of different crude extract, depicting variable total phenolic content, total flavonoid content, antioxidant activity*, and EC_50_ values.

Name of crude extract	Antioxidant activity (%)	EC_50_ values (*μ*g/mL)	Total phenolic (mg GAE/g dw)	Total flavonoid (mg QE/g)	Total phenolic/Total flavonoid
HE I	28.16 ± 4.76^c^	ND	11.35 ± 0.26^ab^	0.45 ± 0.49^a^	25.22
CE I	0.46 ± 0.98^a^	ND	72.10 ± 0.71^g^	53.44 ± 0.94^e^	1.35
EAE I	4.10 ± 0.14^a^	ND	41.82 ± 1.34^e^	11.57 ± 1.07^c^	3.61
ME I	69.51 ± 1.03^f^	81.32	9.91 ± 0.33^a^	ND	ND
ME II	46.99 ± 4.69^d^	121.90	13.76 ± 1.52^b^	ND	ND
CE II	47.16 ± 4.98^d^	215.30	35.02 ± 0.11^d^	60.14 ± 0.33^f^	0.58
DE II	60.69 ± 2.46^e^	373.50	54.48 ± 1.65^f^	21.84 ± 1.65^d^	2.49
EAE II	8.62 ± 0.95^a^	ND	40.76 ± 2.22^e^	7.58 ± 0.23^b^	5.38
BE II	18.34 ± 2.34^b^	ND	22.47 ± 2.03^c^	ND	ND

Each value represented the mean ± SD of three replicates (*n* = 3). Values with different letters are significantly different(*P* < 05.)based on One Way and Tukey HSD test.

Data of total phenolic contents are expressed as milligrams of gallic acid (GAE) equivalents per microgram dry weight.

Quercetin and BHT were used as a positive control, EC_50_= 4.95 *μ*g/mL and 50.31 *μ*g/mL and the final concentration were 125 *μ*g/mL.

ND = Not determined

*Modarresi Chahardehi et al. [9]

**Table 2:** Antimicrobial activity of *Pilea microphylla. *

Pathogenic microorganism	HE I	CE I	EAE I	ME I	ME II	CE II	DEE II	EAE II	BE II
Bacteria									
* Acinetobacter calcoaceticus*	−	−	−	−	−	−	−	−	−
* Bacillus cereus*	+	+	+	+	−	−	−	−	+
* Bacillus cereus ATCC 10876*	+	−	−	−	−	+	−	−	+
* Bacillus subtilis*	−	−	−	−	−	+	−	+	+
* Bacillus licheniformis ATCC *	−	−	−	−	−	−	−	−	−
* Bacillus spizizenii ATCC 6633*	+	++	−	+	−	−	−	−	−
* Citrobacter freundii*	+	−	+	−	−	+	−	−	+
* Entrobacter aerogenes*	−	−	−	−	−	+	−	−	−
* Escherichia coli*	−	−	−	−	−	+	−	−	−
* Erwinia *sp.	+	+	−	++	−	+	−	−	+
* Klebsiella pneumoniae*	−	−	−	−	−	−	−	−	−
* Klebsiella pneumonia ATCC13883*	−	−	−	−	−	−	−	−	−
* Micrococcus *sp.	−	−	+	−	−	++	−	−	−
* Morganella morganii*	−	−	−	−	+	−	−	−	−
* Methicillin-resistant *Staphylococcus aureus *(MRSA)*	−	−	−	−	−	+	−	−	−
* Pseudomonas aeroginosa ATCC 27853*	−	−	−	−	−	−	−	−	−
* Pseudomonas stutzeri ATCC 17588*	−	−	−	−	−	−	−	−	−
* Salmonella paratyphi B*	−	−	−	−	−	+	−	−	+
* Serratia marcenes*	−	−	−	−	+	+	−	−	−
* Shigella boydii ATCC 9207*	−	−	−	−	−	−	−	−	−
* Staphylococcus aureus*	−	−	−	−	−	−	−	−	−
* Staphylococcus aureus ATCC 12600*	−	−	−	−	−	−	−	−	−
* Staphylococcus epidermidis ATCC 1228*	−	−	−	−	−	−	−	−	−
* Streptococcus salivarius ATCC 13419*	−	+	−	+	−	−	−	−	−
* Vibrio parahaemolyticus*	−	+	−	−	−	−	+	−	−
* Yersinia *sp.	−	−	−	−	−	+	−	−	+

Yeast									
* Candida albicans*	−	−	−	−	−	−	−	−	−
* Candida utilis*	−	−	−	−	−	−	−	−	−
* Sacharomyces servisiae*	−	−	−	−	−	−	−	−	−

Fungi									
* Aspergillus fumigatus*	−	−	−	−	−	−	−	−	−
* Aspergillus niger USM AI1*	−*	−	−	−	−	−	−	−	−
* Rhizopus *sp.	−	−	−	−	−	−	−	−	−
* Trichophyton rubrum*	−	−	−	−	−	−	−	−	−

Antimicrobial activity based on the diameter of inhibiotion zone (mm) follow this skim: ++10–14 mm, + ≤ 9 mm.

*Hexane extract showed reduced of inoculum but not hypha.

**Table 3:** In vitro activity of pathogenic bacteria for MIC and MBC values (mg/mL).

Bacteria	Crude plant extract	MIC value (mg/mL)	MBC value (mg/mL)
*Bacillus cereus*	BE II	33.33	33.33
*Bacillus spizizenii ATCC 6633*	EAE I	8.33	8.33

**Table 4:** The LC_50_ values of Percentage lethality of *Artemia salina *(*μ*g/mL).

Crude plant extracts	LC_50_		
	6 hrs	12 hrs	24 hrs
HE I	—	3943	3880
CE I	2836	183	2
ME I	2840	183	16
CE II	2836	542	79
BE II	2840	545	194

— = Not converge.

## Abbreviations

HE I: hexane extract of method I,
CE I: chloroform extract of method I,
EAE I: ethyl acetate extract of method I,
ME I: methanol extract of method I,
ME II: methanol extract of method II,
CE II: chloroform extract of method II,
DEE II: diethyl ether extract of method II,
EAE II: ethyl acetate extract of method II,
BE II: butanol extract of method II.
